# Nanostructured Surfaces of Dental Implants

**DOI:** 10.3390/ijms14011918

**Published:** 2013-01-17

**Authors:** Eriberto Bressan, Luca Sbricoli, Riccardo Guazzo, Ilaria Tocco, Marco Roman, Vincenzo Vindigni, Edoardo Stellini, Chiara Gardin, Letizia Ferroni, Stefano Sivolella, Barbara Zavan

**Affiliations:** 1 Department of Neurosciences, University of Padua, Padua 35133, Italy; E-Mails: eriberto.bressan@unipd.it (E.B.); sbricoli.luca@gmail.com (L.S.); riccardo.guazzo@gmail.com (R.G.); ilaria.toccotussardi@gmail.com (I.T.); vincenzo.vindigni@unipd.it (V.V.); edoardo.stellini@unipd.it (E.S.); stefano.sivolella@libero.it (S.S.); 2 IDPA-CNR, Institute for the Dinamics of Environmental Systems Calle Larga S. Marta 2137, Venice 30123 (VE), Italy; E-Mail: marco.roman@unive.it; 3 Department of Biomedical Sciences, University of Padua, Padua 35133, Italy; E-Mails: chiara.gardin@unipd.it (C.G.); letizia.ferroni@gmail.com (L.F.)

**Keywords:** adult stem cells, nanotechnologies, differentiation, osteogenesis, surfaces, dental implant

## Abstract

The structural and functional fusion of the surface of the dental implant with the surrounding bone (osseointegration) is crucial for the short and long term outcome of the device. In recent years, the enhancement of bone formation at the bone-implant interface has been achieved through the modulation of osteoblasts adhesion and spreading, induced by structural modifications of the implant surface, particularly at the nanoscale level. In this context, traditional chemical and physical processes find new applications to achieve the best dental implant technology. This review provides an overview of the most common manufacture techniques and the related cells-surface interactions and modulation. A Medline and a hand search were conducted to identify studies concerning nanostructuration of implant surface and their related biological interaction. In this paper, we stressed the importance of the modifications on dental implant surfaces at the nanometric level. Nowadays, there is still little evidence of the long-term benefits of nanofeatures, as the promising results achieved *in vitro* and in animals have still to be confirmed in humans. However, the increasing interest in nanotechnology is undoubted and more research is going to be published in the coming years.

## 1. Introduction

Implant dentistry has undergone a slow but steady growth during the last 30 years. When teeth are missing for caries, periodontal disease or agenesis, dental implants, such as dentures, are used either to replace the missing elements or to support complex prostheses. The surgical procedure is mostly standardized: the recipient bone site is prepared with precision drills and then the titanium screw is screwed into place at precise torque and speed, and consequently at standardized time. Subsequently, some months are generally needed before placing a definitive restoration. Time is fundamental to achieve osseointegration.

When the idea of osseointegration first emerged in the late 1970 and 1980 [1,2], nobody thought dental implants would have had a real breakthrough over the years. Osseointegration actually refers to a structural and functional fusion of the implant surface with the surrounding bone. This is thought to be thoroughly important for the short and long term success of the dental implant, and is strictly related to the geometry and surface topography of the device, not forgetting the surgical technique of implantation. This is the reason why the interest in dental implant technology is increasing. The devices are designed to mimic as much as possible cell interactions that normally take place during bone remodeling. In this context, synthetic materials should have surface features as similar as possible in diameter and shape to the bone, in order to increase the rate and extent of osseointegration. The features of the implant surface can be characterized at different size levels.

At a macroscopic level, the screws design, the thread shape and the pitch distance are fundamental to give stability to implants. As postulated by Abuhussein *et al*. [3], dental implants should be designed to maximize favorable stresses and to minimize undesirable stresses along the bone-implant interface. Specifically, the use of a smaller pitch, deeper threads, and longer and larger implants may be of help, increasing the surface area in contact with the surrounding bone.

The microscopic level involves the coating of the implant surface. *In vitro* [4] and *in vivo* [5,6] studies have been conducted to assess the modifications in the bone-implant interactions brought by these surface modifications. Microscale features are generally believed to create a microenvironment that can modulate cells recruitment and function [7]. In particular, authors proved that the roughness of the surface can influence osseointegration by mean of cell attraction, improving cells adhesion [8–10]. However, the role of the microscopic features of the implant surface on bone formation at the implant site is believed to be indirectly involved in the osteointegrations process [11–13].

Nowadays, the improvement of the bone-forming activity at the bone-implant interface is committed to nanoscale features that have the ability to induce the differentiation of stem cells along the osteogenic pathway [7]. Nanotechnology involves the creation of functional materials, devices and systems through control of matter on the nanometer length scale (1–100 nm). The application of nanotechnology to biomedical surfaces is explained by the ability of cells to interact with nanometric features. The biological effect is mainly mediated by integrins, binding to the arginine-glycine-aspartate sequences of peptides. Cells adhesion to the extra cellular matrix (ECM) causes clustering of integrins into focal adhesion complexes (FA), and activates intracellular signaling cascades [14]. In this context, nanofeatures are crucial to modulate stem cells behavior [15]. The surface pattern in particular has been demonstrated to play a key role, as osteoblasts are able to “encode” the three-dimensional characteristics of the surface (e.g., lines, pores, dots) and modulate their growth according to the suggested structural features [4,10,11]. Nanoscale dimensional patterns are so crucial that a slight modification in the nanotube diameters is able to cause the transition from cells adhesion and spreading enhancement (observed for 15–30 nm TiO_2_ nanotubes) to growth decay (≥50 nm diameter) [16]. Cavalcanti-Adam *et al.* [17] confirmed this observation, demonstrating that 58 nm spacing promoted FA formation, while 108 nm spaces failed to stimulate FA development.

Given the importance of nanofeatures of dental implants surfaces in the achievement of osseointegration, we provide a review article of the most common manufacture techniques and the related cells-surface interactions.

## 2. Cell and Surface

Stem cells (SCs) are generally defined as cells that are able to self-renew and to differentiate into various specialized tissues (e.g., fat, bone and cartilage, neural cells) [18]. Their main functions are tissue development, homeostasis and in the case of tissue damage, reparation. Multipotent mesenchymal stromal cells (MSCs) were found to be rare cells living in various mesenchymal tissues, for example in the bone marrow stroma, adipose tissue, dental pulp. Currently MSC represents an innovative tool in regenerative medicine and odontoiatric field stem cell biology is now is fulfilling tools for the development od biomedical devices for bone or tooth restoration [19]. Indeed, current dentistry resolves the problems related to those loose using autologous tissue grafts or metallic implants, but these treatments have some limitations such as an adjoining tooth damage, bone resorption *etc.*

Cell therapies represent the most challenging and, potentially, the most successful application of stem cells (SCs). Because of their ability to differentiate into different types of functional cells, SCs posses great potential in therapeutics to restore and regenerate native tissues. Atypical strategy based on SCs consists in engineering tissues by using cells coupled with suitable biomaterials to mimic the *in vivo* biochemical and biophysical microenvironment [19]; this approach has shown promising results in treating irreparable damage of native tissues caused by diseases or injuries [20]. However, before SC-based therapies are applied in clinics, a fundamental issue needs to be elucidated to gain a precise control over SCs response, in terms of self-renewal and differentiation, specifically, a broader understanding of the interplay between SCs, the surrounding microenvironment components (growth factors, cell-cell contacts, and cell-extracellular matrix interactions), and external forces [21], which is currently lacking [22].

In this view, the most applied stem cells are Human mesenchymal stem cells (hMSCs) that are self-renewing cells with multipotent differentiation potential.

They give rise to various anchorage-dependent cell types, including adipocytes, chondrocytes, myoblasts, and osteoblasts. Their differentiation potential is influenced by substrate elasticity, geometrical connement, and substratetopography [23].

Cell-substrate or cell-extracellular matrix (ECM) adhesions are mediated by dynamic multiprotein structures called focal adhesions (FA). They are important for force transmission, cytoskeletal regulation and signaling. At these sites, the cell establishes a transmembrane connection between elements of the ECM and the actin cytoskeleton. The transmembrane integrin proteins orchestrate these events [24]. The integrins, heterodimers containingalfa and beta subunit, bind with their extracellular domain to the ECM proteins fbronectin, laminin, and vitronectin.

The cytosolic domain of integrins binds to a large number of proteins such as paxillin and zyxin either directly or via scaffolding proteins. Some of these proteins are implicated in strengthening the linkage between the extracellular matrix and the cytoskeleton, others play a role in adhesion-mediated signaling. Cellular adhesions can be classified into three categories: Focal complexes (FX), FA and fibrillar adhesions. The FX along the leading lamella of migrating cells are early adhesions, which transform into focal adhesion upon RhoA activation [25,26] or as a result of external mechanical perturbation [27,28]. Fibrillar adhesions develop from FAs following actomyosin contraction. Recruitment of zyxin protein has been proposed as a molecular marker for mature Fas. Zyxin facilitates actin polymerization in response to mechanical forces and dissociates from focal adhesions upon force dissipation [29]. Focal adhesions are closely linked to cellular migration, which is driven by repeated cycles of protrusion of the leading edge, formation of new matrix adhesions and retraction of the trailing edge. FA play a dual role in motility. On one hand, they provide a robust anchor to the ECM, necessary for the actomyosin system to exert the force to pull the cell body and the trailing edge forward, but on the other hand they may also restrain the migration process. The mechanical connections between the matrix and the cytoskeleton allow cells to exert traction forces that are transmitted to the cell nucleus through intracellular pathways; the resulting force triggers signaling transduction into biochemical signals that affect SC response, for example, the synthesis of specific transcription factors in the nucleus. Various mechanotransduction pathways have been proposed, including the Ras/MAPK, the PI3K/Akt, RhoA/ROCK, Wnt/catenin, and the TGF-*beta* pathways, which are generally integrin-based, and mechano-sensitive ion channels.

Recent studies have shown that mechanical cues, including the stiffness of the substrate, the nanotopography of the adhesion surface, and extracellular forces, are able to direct stem cell fate *in vitro*, even in the absence of biochemical factors [30].

## 3. Stem Cells and Bone Commitment

Osteogenesis is an active process tightly regulated to ultimately generate a normal vascularized bone structure. Bone formation depends on the cooperation of several factors, namely: (i) the genesis of specific cell types such as progenitor cells and osteoblasts; (ii) a mineralized extracellular matrix scaffold; (iii) soluble bioactive molecules (cytokines, growth factors, hormones, ions, vitamins); and (iv) mechanical stimuli. In the adult, the osteoblast is derived from a bone marrow stromal fibroblastic stem cell termed the mesenchymal stem cell (MSC), a non-hematopoietic multipotent stem-like cell vital for the osteogenic process and capable of differentiating into both osteoblastic and non-osteoblastic lineages [31].

The commitment and differentiation of MSCs towards osteogenic lineage is regulated by a certain group of factors. Among these factors, the initial and most specific marker is Runx2. Runx2 activates and regulates osteogenic differentiation by two independent signaling pathways via transforming growth factor-beta 1 (TGF β1) and bone morphogenetic protein 2 (BMP2) [32].

Along with Runx2, BMP2 and distal-less homeobox 5 (Dlx5) commit MSCs towards the osteogenic lineage. Commitment is the process that restricts MSCs to respond and undergo differentiation towards a specific lineage. In addition to the induction of osteogenic differentiation, Runx2 inhibits the differentiation of MSCs towards the adipogenic lineage. BMP2 induces the expression of Osx independent of Runx2 [33].

Following commitment, MSCs are differentiated into preosteoblasts. Preosteoblast are elliptical in shape with an elongated nucleus and are capable of proliferation. They express Runx2, D1x5, msh homeobox homologue 2 (Msx2), P2Y4 and P2Y14 [34], and few markers of osteoblasts such as ALP, type I collagen, and osteopontin (OPN), but their expression is weaker than immature osteoblasts. Alkaline phosphatase is one of the early proteins and regulates bone mineralization.

β-Catenin, Runx2, and Osx differentiate preosteoblasts into immature osteoblasts. These cells are spindle shape. They express bone matrix protein, bone sialoprotein, and OPN [35].

At later stages, Runx2 inhibits the maturation of osteoblasts. Osx causes the terminal maturation of osteoblasts and induces osteocalcin expression. When osteoblasts are completely differentiated, they become cuboidal and produce a self-mineralized organic matrix. The expression of OPN is reduced in mature osteoblasts; while the expression of other proteins such as P2X5, alkaline phosphatase, collagen type I, and osteocalcin [36] is increased.

## 4. Osseointegration of Dental Implants

Biomaterials are never truly inert, being at best biotolerable. The cell-substratum interface functions as more than a simple boundary of definition between the host and an implanted device, rather it presents primary cues for cellular adhesion and subsequent induction and tissue neogenesis. Indeed, the function and cytocompatibility of a construct can be assessed *in vitro* by observing the viability and adhesion of cells at the substratum interface [37]. The range of materials currently in use within biomedical applications and their lack of biofunctionality reflects an increasing need for biomimetic constructs but also indicates the challenges present within the field, *i.e.*, to ultimately control the interactions that occur at the cell-substratum interface [38].

A key tenet of medical device technology is to use the exquisite ability of biological systems to respond to the material surface or chemical stimuli in order to help develop next-generation biomaterials [39]. In order to investigate the reaction elicited by a material *in vivo* an understanding is required of the roles played by the cytoskeleton, cellular membranes, and the extracellular matrix (ECM) following implantation of a foreign material [40]. An increased knowledge of the extracellular environment, topographical and chemical cues present at the cellular level and how cells react to these stimuli has resulted in the development of advanced orthopedic materials with an aim to regulate cell attachment and subsequent cellular function [38].

In recent years it has become self-evident that cells can use features such as filopodia (or microspikes), which have a tip diameter in the range of 50–100 nm, to gather and use spatial information. The cells can use filopodia to produce contact guidance with features as small as 10 nm high—around the size of a typical protein [40]. In addition, it has also been observed that MSCs have an increased interaction with topography compared with differentiated cells like fibroblasts. This evidence that stem cells are exquisitely sensitive to their nanoenvironment adds further evidence that the topographical environment is important for tissue-specific differentiation [41]. The recruitment of immunological cells to a site of implant involves a complex cascade of immune mediators, including various cell types, soluble signaling molecules, and cell-cell interactions. Previous studies have made it clear that the macrophage is the dominant cell in the foreign body response. Once adhered to an implanted material single macrophage cells fuse through a complex series of events to form multinucleated giant cells; this response is accompanied by the recruitment of fibroblasts and fibrous tissue formation. The adherence of giant cells to a biomaterial surface is correlated to the release of enzymes (e.g., esterases, lipases) and other bioreactive intermediates that can degrade and cause a loss of implant function [37]. It follows that the regulation of cellular adhesion or selective adhesion of specific cellular phenotypes is crucial to regulate optimal tissue-specific integration while preventing inflammatory cell recruitment and scar tissue formation.

Conversely, inert materials may be successfully employed for applications in which protein and/or cellular interaction may reduce device functionality. *In vitro* studies indicate that endogenous proteins become rapidly adsorped to a material surface providing a structural framework on which cellular adhesion may initiate. Modern implants make use of chemical and topographical modification to regulate cellular adhesion [38], differentiation, and *de novo* tissue deposition.

Therefore, it should to put in evidence that that more cues for the cell destiny come from additional parameters, such as the stiffness of the substrate [42], its chemical composition [43], its availability to mineralization [44,45] presence of proper extracellular matrix [46,47] and many other parameters.

In particular, recent developments in small technologies encompassing the generation of micro- and nanoscale structures have been successfully translated into the development of second generation implantable materials. Strategies adopted are described in the following sections.

## 5. Surface Modifications

The application of nanotechnology to dental implant surfaces deals with many different arrangements. In particular, surfaces could potentially assume an organized (isotropic) or unorganized (anisotropic) pattern. Due to the difficulties of application of standardized sequences to complex designs, the pattern for dental implants is generally anisotropic [48].

A great variety of techniques are used to create nanofeatures on dental implants surface. These can be divided into chemical and physical processes.

### 5.1. Chemical Modifications

#### 5.1.1. Anodic Oxidation

**Figure 1:**
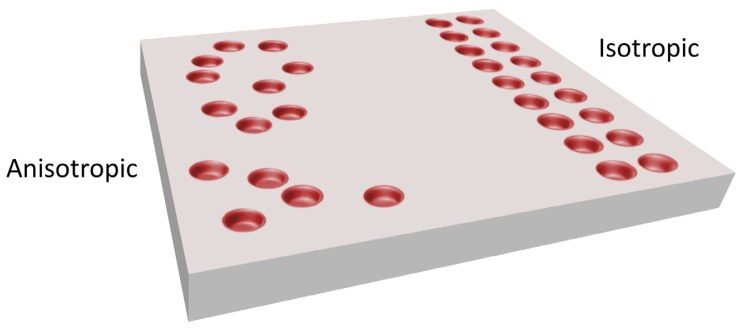
Difference between isotropic (uniform in all direction) and anisotropic distribution of surface nanofeatures. Due to the complexity of dental implants design these are usually anistropic.

Anodization is one of the most commonly used techniques to create nanostructures with diameters of less than 100 nm on titanium implants [49]. Voltage and direct current (galvanic current) are used to thicken the oxide layer among the implant surface. The titanium substrates serves as the anode in the process, while an inert platinum sheet provides the cathode. The anode and cathode are then connected by copper wires and linked to a positive and negative port of a 30 V/3 A power supply, respectively. During the process, the anode and cathode are kept separated (about 1 cm distance) and they are submerged into an electrolyte solution in a Teflon beaker. Diluted hydrogen fluoride (either at 0.5 wt% or 1.5 wt%) is used as electrolyte. Subsequently, a strong acid dissolves the oxide layer creating a pattern that follows the convective lines of the galvanic current. Therefore, through the regulation of voltage and density it is possible to control the diameters of nanotubes and the gap between them (Figure 1).

**Figure 2:**
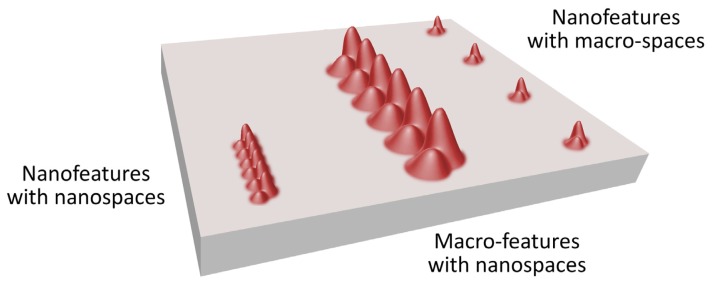
Characterization on nano-surfaces: nanoscale features separated by nanoscale spaces; microscale features separated by nanoscale spaces; nanoscale features separated by microscale spaces.

As an example, the outcome of the anodization of titanium in diluted hydrofluoric acid at 20 V for 20 min is the creation of surface nanotubes, while the anodization at 10 V for the same time produces nanoparticles. In addition, the distance between nanotubes/nanoparticles can be very different among different surfaces. Nanoscale features can be separated alternatively by microscale or nanoscale spaces (Figure 2).

Oxidative nanopatterning confers Ti-based metals the exciting capacity to selectively influence cellular behavior by enhancing the growth of osteoblastic cells while limiting the proliferation of fibroblasts. The physic-chemical cueing impacts on gene and protein expression, in a way that is strongly determined by slight modifications of the dimensions of the nanofeatures, as mentioned above [48]. Von Wilmowsky *et al.* provided the confirmation that implant surface with interface features of 30 nm TiO_2_ nanotubes positively influence bone-to-implant contact (BIC) and peri-implant bone formation [50].

#### 5.1.2. Combinations of Acids (Bases) and Oxidants

The combination of strong acids is effective in creating a thin grid of nanopits on a titanium surface (diameter 20–100 nm) [48]. The titanium sample etched with a solution of strong acids, e.g., H_2_SO_4_ and H_2_O_2_, at a constant temperature and for a specific duration. Etching is then stopped by adding distilled water. The recovered disks are washed further with ethanol in an ultrasonic bath for 20 min and dried [51].

**Figure 3:**
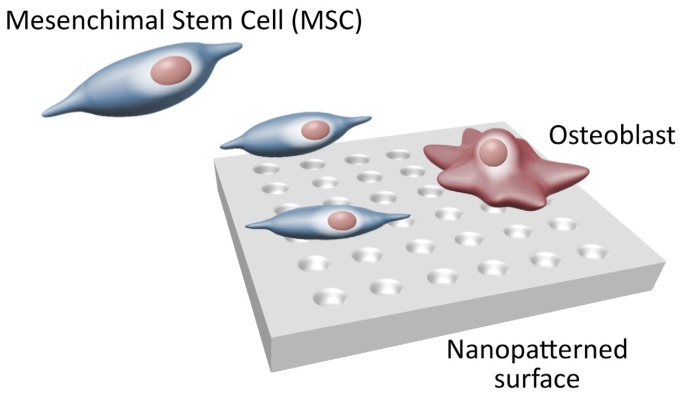
Mesenchimal Stem Cells (MSCs) Differentiation Process. Nanostructured Surfaces promote MSCs osteogenic differentiation while limiting fibroblast differentiation.

As for anodic oxidation, some reaction parameters such as temperature, duration, and solutes, can be adjusted in order to modify the number and depth of nanopits, therefore modulating cell function. Specifically, the treatment with H_2_SO_4_-H_2_O_2_ on titanium screw-shaped implants creates a nanopattern that has been demonstrated *in vivo* to be associated with an enhanced osteogenesis [48]. Vetrone *et al.* [52] confirmed the observation, stating the promotion of stem cells growth provided by oxidative nanopattering. Ferreira *et al.* [15] further characterized the most suitable nanoarrangement of TiO_2_ nanotubes, noting that a diameter of 15 nm with a vertical alignment was associated with a high spreading and differentiation of rat mesenchymal stem cells into the osteogenic lineage. Notably, 15 nm roughly correspond to the predicted lateral spacing of integrin receptors in the FA complexes [53] (Figure 3).

### 5.2. Physical Modifications

#### 5.2.1. Plasma Spray

The plasma deposition process is able to create an engineered-surface nanostructure, with features usually standing below 100 nm. First, a vacuum is used to remove all contaminants. Then, kinetic energy guides the charged metallic ions or plasma to the device surface. The process enables a wide range of materials (e.g., Ag, Au, Ti, *etc.*) to be coated onto a wide range of underlying materials (e.g., metals, polymers, and ceramics) [54]. In dental implants, titanium particles deposit on the implant surface with a uniform pattern.

The nanoparticulate coating with titanium particles achieved through the plasma spray technique has been demonstrated to increase the osteoblast density on the implant surface both in *in vitro* and in *in vivo* studies. Particularly, Reising *et al.* [54] detected a greater deposition of calcium on the nano Ti-coated surfaces when compared to uncoated surfaces.

#### 5.2.2. Blasting

Blasting is a technique that leads to the creation of a porous layer on the implant surface achieved through the collision with microscopic particles. The thickness of the porous layer can be modulated by the granulometry of the particles. For example, the surface of commercial endosseous titanium implants is a rough porous layer ranging between 50 and 200 nm created through the combination of particles blasting and hydrogen fluoride treatment [48]. The rough surface has been demonstrated to stimulate osteoblastic gene expression, as well as to enhance bone formation and bone-implant fixation, in a word osseointegration [55,56]. While an associated inflammatory response was reported [57], the overall success rate was satisfactory, with the majority of implants yielding good osseointegration and stability at one year after surgery [48].

Among the range of available materials, aluminia is one of the most used for blasting. Nevertheless, Aparicio *et al.* [58] highlighted some features related to alumina blasting for dental implants that could compromise osseointegration, like particles detachment during the healing process and absorption by the surrounding tissues.

TiO_2_ is also used as a blasting material showing interesting results in experimental studies. Particularly, TiO_2_ blasted implants were associated in humans to a significant enhancement of BIC when compared with machined surfaces [59]. This result was confirmed by Rasmusson *et al.* [60] who investigated the osteogenic properties of titanium grit-blasted surfaces.

A further enhancement in the blasting technology was achieved through the integration of bioceramic grit-blasting and acid etching (BGB/AE), to produce submicrometric topographies on titanium implants. The evaluations made two months after implantation showed a significantly higher BIC and osteocyte density around modified implants when compared to simple dual-acid etching implants [48]. Clinically, the combination of blasting and etching on the surfaces has been associated to a 10-year cumulative survival rate of 96.2% [61]. Not surprisingly, Masaki *et al.* [62] further demonstrated that around this surface human mesenchymal stem cells increased the expression of type I collagen and of alkaline phosphatase, which is a key enzyme in the biomineralization along the bone-implant interface.

## 6. Conclusions

In this paper, we stressed the importance of the modifications on dental implant surfaces at the nanometric level. Nowadays, there is still little evidence of the long-term benefits of nanofeatures, as the promising results achieved *in vitro* and in animals have still to be confirmed in humans. Additionally, there is a lack of data about the release of metal ions in the surrounding tissues and the possible systemic effects. Moreover, a complicating feature of nanoscale manipulation is that there are many chemical changes on the bulk material surface and it can be very difficult to investigate positive or negative effects induced [63]. However, the increasing interest in nanotechnology is undoubted and more researches are going to be published in the next years. Ongoing developments suggest that dental implant manufacturers will invest increasing resources to give patients the most durable and most biocompatible material to replace their teeth.
